# Dye-sensitized photocatalyst for effective water splitting catalyst

**DOI:** 10.1080/14686996.2017.1375376

**Published:** 2017-10-09

**Authors:** Motonori Watanabe

**Affiliations:** ^a^ International Institute for Carbon-Neutral Energy Research (I2CNER), Kyushu University, Fukuoka, Japan

**Keywords:** Photocatalytic water splitting, hydrogen production, dye-sensitized, Z-scheme: organic–inorganic composite, 50 Energy Materials, 205 Catalyst / Photocatalyst / Photosynthesis

## Abstract

Renewable hydrogen production is a sustainable method for the development of next-generation energy technologies. Utilising solar energy and photocatalysts to split water is an ideal method to produce hydrogen. In this review, the fundamental principles and recent progress of hydrogen production by artificial photosynthesis are reviewed, focusing on hydrogen production from photocatalytic water splitting using organic–inorganic composite-based photocatalysts.

## Graphical abstract

**Figure:**
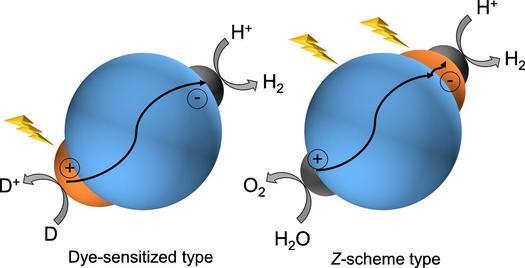

## 1. Introduction

Renewable hydrogen production is key for establishing clean energy systems because hydrogen can be used as a clean energy source in hydrogen fuel cells and a hydrogen-driven society [1,2]. However, the only practical hydrogen production source at present is fossil fuels [3]. Fossil fuels are finite and burning them releases carbon dioxide, which has impacts on the global environment such as global warming. To accomplish renewable energy production, a novel hydrogen production method is required. The ideal source is water. Water can be split into hydrogen (H_2_) and oxygen (O_2_) through chemical reactions including electrolysis, biomass, thermal, thermochemical and photochemical reactions, among which electrolysis is the most practical method nowadays. In the early 2000s, 95% of hydrogen production was achieved by reforming fossil fuels, and 5% was obtained from water by electrolysis [3]. Biomass, thermal and thermochemical processes are still in the demonstration stage. On the other hand, hydrogen production using solar energy with photocatalysts is an ideal ‘holy grail’ generation method because: (i) it only involves a simple and clean reaction that directly converts solar energy to hydrogen; (ii) only a photocatalyst, sunlight and water are required; (iii) the reaction occurs under mild conditions (even at room temperature); and (iv) water is split into hydrogen without producing carbon dioxide as a by-product. Although this method has many environmental advantages, many challenges must be overcome before photocatalytic water splitting can be practically implemented. The most challenging issues with photocatalysts are how to take advantage of light beyond the ultraviolet (UV) region and how to improve charge extraction in photosynthesis [4−11]. A photocatalyst that can utilize visible light would be more effective because UV light accounts for only 5–7% of sunlight, whereas visible light accounts for 47%, and the remaining energy falls in the near-infrared (IR) region of the electromagnetic spectrum [12]. Here, we focus on semiconductor-based photocatalysts and their photocatalytic water-splitting performance. One method for using visible light is to employ organic semiconductors with reduced band gaps compared with conventional inorganic semiconductors to obtain the driving force for charge separation in photosynthesis. Therefore, various types of organic–inorganic composites for hydrogen production by water splitting are reviewed herein.

## 2. Mechanism of photocatalytic water splitting

The first example of water splitting was demonstrated by Honda and Fujishima [13]. They conducted water splitting using a rutile titanium oxide (TiO_2_) electrode as the anode and a platinum (Pt) electrode as the cathode in an electrochemical cell system in 1972. When the photon energy was absorbed by semiconducting TiO_2_, electron–hole pairs were separated at the interface between TiO_2_ (hole) and Pt (electron). When the light is irradiated on TiO_2_, the absorbed light energy makes it possible to decompose water into hydrogen and oxygen. This phenomenon is called the Honda–Fujishima effect [13]. Bard reported a photocatalysis system using Pt-loaded TiO_2_ powders for water splitting in 1980 [14]. The success of this system indicates that cell-type systems such as a TiO_2_–Pt system [15] are not required for photocatalytic water splitting, and photocatalytic reactions can proceed with powder-type photocatalysts, such as a TiO_2_–ruthenium oxide (RuO_2_) powder system [16]. These findings have inspired many scientists to investigate hydrogen gas production by photocatalytic water splitting.

**Figure 1.**
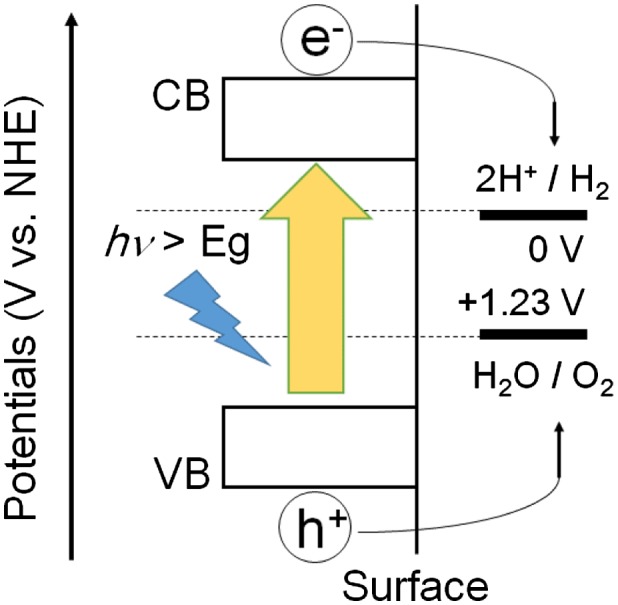
Schematic of photocatalytic water splitting. NHE: the normal hydrogen electrode. CB: conduction band. VB: valence band. Eg: band gap.

The mechanism of photocatalytic water splitting is shown in Figure 1. First, when the optical band gap (Eg) in the photocatalyst is irradiated, electrons in the valence band are excited to the conduction band (CB), and holes are generated in the valence band (VB). The photo-generated charges then move to the surface of the catalyst, the electrons at the conduction band reduce protons (H^+^) to generate hydrogen while water is oxidized by the holes at the valence band to produce oxygen. The hydrogen production reaction requires a potential more negative than 0 V vs the standard hydrogen electrode (SHE) for the H^+^/H_2_ half-reaction (pH = 0), and oxygen production requires a potential more positive than 1.23 V vs SHE for the O_2_/H_2_O half-reaction (pH = 0). The reactions involved in water splitting can be expressed by equations (1, 2).(1)
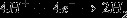

(2)
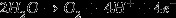



In the case of powder-type photocatalysts, electron–hole pairs are produced after light absorption. Almost all electrons and holes recombine before photochemical reactions occur. For example, Bahnemann et al. [17] reported that 80% of charge carriers recombined within 200 ns in 2.4 nm TiO_2_ particles after 10 mJ/pulse band-gap excitation, and Serpone et al. [18] found that 100% recombined within 10 ns in 2.1 nm TiO_2_ particles and 90% in 13 nm TiO_2_ particles after 2.5 mJ/pulse band-gap excitation. The unrecombined electrons and holes are transported to the surface, and water is decomposed to hydrogen and oxygen by electrons and holes, respectively. In the case of TiO_2_ particles, Tang et al. [19] reported that free holes and electrons are generated picoseconds after excitation, and the hydrogen production reaction occurs on the order of microseconds. The water oxidation reaction is much slower than the hydrogen production reaction because the former reaction requires four electrons to be transferred. These results suggest that charge extraction plays an important role in photocatalytic water splitting.

## 3. Visible-light-driven semiconductor materials as photocatalysts

In the sunlight spectrum (AM 1.5), the UV region (280–380 nm) is narrower than other regions [12]. The UV component of sunlight is only 5–7%, whereas the visible-light (VL) contribution is 47%. If VL could be used, the efficiency of water splitting would improve dramatically. However, one of the key issues for water splitting is the difficulty in using visible light.

**Figure 2.**
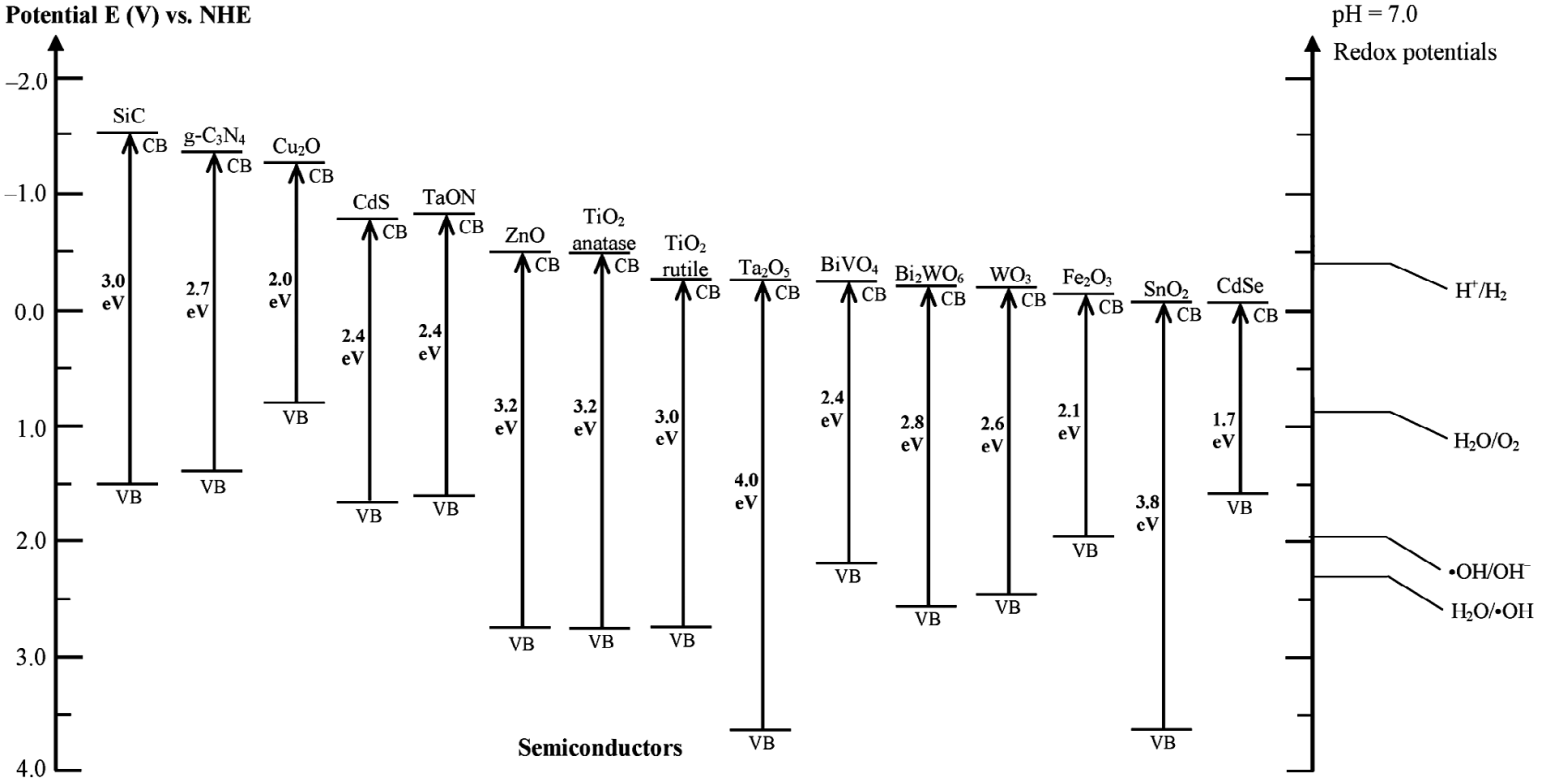
Energy potentials of semiconductors and redox energies for hydrogen production and water oxidation at pH = 7. Reprinted with permission from Ref. [20]. Copyright 2016 American Chemical Society.

Typical photocatalysts are presented in Figure 2 [20]. Metal oxide semiconductors that are stable and easy to synthesize, such as tungsten oxide (WO_3_), bismuth vanadium oxide (BiVO_4_) and tin dioxide (SnO_2_), can be potential candidates for visible-light-driven photocatalysts. The band gaps of WO_3_ [21, 22] and BiVO_4_ [23, 24] are 2.8 eV (440 nm) and 2.4 eV (517 nm), respectively; however, they could not be used for hydrogen production due to their deep conduction band (CB) levels at potentials more positive than 0 V vs NHE (pH = 0). Several visible-light-responsive photocatalysts have been reported [4–6]. Non-oxide semiconductor of cadmium sulfide (CdS, 2.4 eV, 517 nm) showing a visible-light-responsive property has an ideal band level for water splitting; however, cadmium is toxic and decomposes during photoreaction [25–27].

The valence band (VB) of oxynitrides is higher than that of the corresponding metal oxides, whereas the conduction band (CB) of oxynitrides is almost not affected to bottom down the potential due to p - d repulsion [5]. Oxynitrides such as TaON [28−30], Ta_3_N_5_ [31,32] and LaTiO_2_N [33−35] show visible light responses and are remarkably stable when used as photocatalysts for water splitting. Solid solutions have also been used as visible-light-responsive photocatalysts [36−39]. For example, solid solution nanocrystals of gallium nitride−zinc oxide (GaN:ZnO, band gap of 2.6 eV) with a co-catalyst was demonstrated for water splitting under visible-light irradiation at 420–440 nm [40]. Although some semiconductors exhibited high visible-light-driven photocatalytic activity, challenges still remain such as improving the water-splitting hydrogen production efficiency and the stability of catalysts by semiconductor engineering [41−43].

Dye-sensitized photocatalytic water-splitting systems (dye–inorganic semiconductor) may be an alternative for achieving hydrogen production under visible light irradiation. By using the visible-light absorption of dye molecules, photo-excited electrons can be efficiently transferred to inorganic semiconductors, and hydrogen can be produced using these electrons. Since the dye molecules are used as the light absorber, the inorganic semiconductors only need to be UV-responsive materials (>3.0 eV). As it is possible to separate charges from the dye to the inorganic semiconductor over a long distance, charge separation is easier than that in a photocatalyst. In addition, since the photon energies absorbed can be changed by modification of the dye molecule, various inorganic semiconductors can be used in dye-sensitized photocatalytic water-splitting systems.

**Figure 3.**
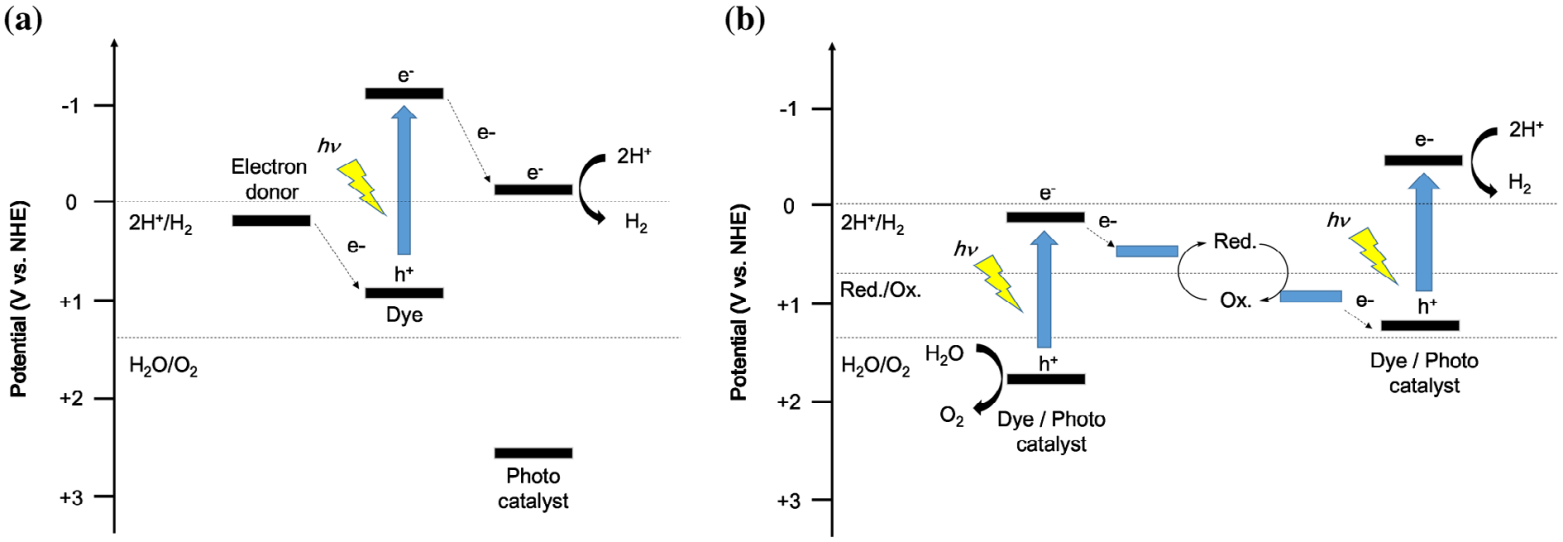
Two types of water-splitting system using organic–inorganic composites. (a) Dye-sensitized photocatalysts for hydrogen production and (b) Z-scheme-type photocatalysts for hydrogen and oxygen production.

For organic–inorganic composite photocatalysts, charge transfer can be controlled by a combination of materials. Figure 3 shows two types of organic–inorganic composite photocatalysts. The mechanism of dye-sensitized photocatalysts is generally similar to that of dye-sensitized solar cells. The dye reaches the excited state by photoexcitation. After that, electrons are injected from the excited state of oxidation potential of the dye to the CB of an inorganic semiconductor within a timescale of several hundred femtoseconds [44−47]. The injected electrons travel to the surface of the inorganic semiconductor (or move to the co-catalyst) and react with protons to produce hydrogen (Figure 3(a)).

The other composite system is a Z-scheme photocatalytic reaction system, which mimics the process of plant photosynthesis (PSI and PSII). In the Z-scheme system, electrons that are separated after photoexcitation are transferred from a dye or photocatalyst to another dye or photocatalyst via a mediator. These previously excited electrons can regain high energy by photoexcitation. As a result, the transferred electrons contribute to hydrogen production and the holes contribute to oxygen production (Figure 3(b)).

## 4. Dye-sensitized photocatalysts

### 4.1. Metal complex-based dye-sensitized photocatalysts for hydrogen production

#### 4.1.1. Effects of anchoring groups of ruthenium-complex dyes

A typical dye-sensitized photocatalyst system is fabricated using an organic dye and a metal oxide. TiO_2_ in various phases such as rutile, anatase, P25 and N-TiO_2_ have been used, with different morphologies such as particle, mesoporous and nanotube structures [48]. The CB energy of TiO_2_ (anatase) is −0.5 V vs NHE. Therefore, organic dyes with the excited state of oxidation have potential energies higher that can inject electrons to TiO_2_ (anatase). One advantage of a dye-sensitized photocatalyst system is that it allows us to use more light than visible light even in catalytic systems where the catalysts only absorb ultraviolet light. The first attempt used a system comprising colloidal Pt nanoparticles and RuO_2_ [49−51]. This system can be cycled by methyl viologen (MV) as redox mediator with tris(bipyridine)ruthenium(II) complex system. Ruthenium complexes have been employed as typical sensitizers for dye-sensitized-type hydrogen production in water.

**Figure 4.**
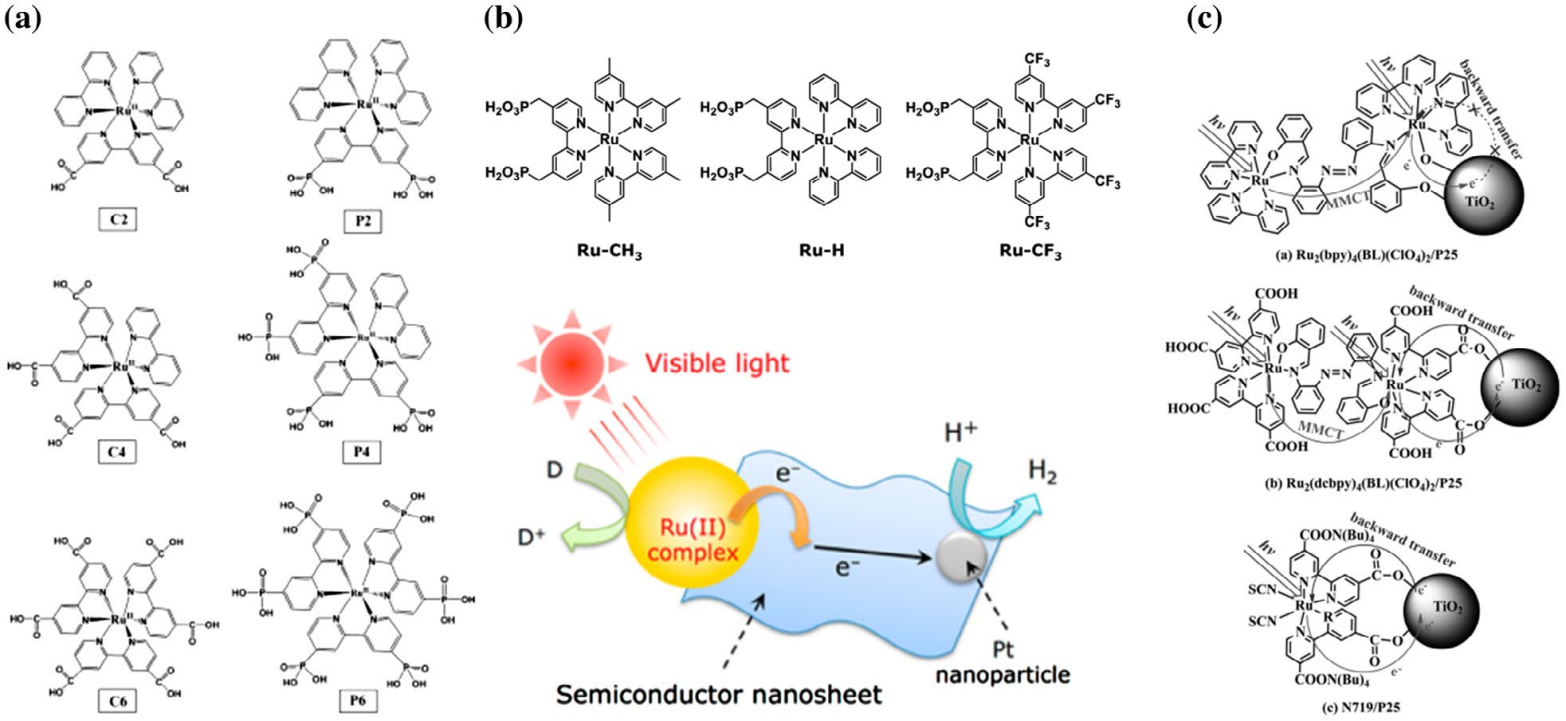
Ruthenium complexes for dye-sensitized photocatalytic hydrogen production. (a) Ruthenium complexes with multiple anchoring groups. Reprinted with permission from Ref. [56]. Copyright 2006 American Chemical Society. (b) Hybrids of a ruthenium(II) polypyridyl complex and a metal oxide nanosheet for hydrogen production. Reprinted with permission from Ref. [58]. Copyright 2015 American Chemical Society. (c) Di-nuclear ruthenium-complex systems for effective dye-sensitized photocatalytic hydrogen production. Reprinted with permission from Ref. [63]. Copyright 2012 John Wiley & Sons.

The structure of the ruthenium complexes can affect hydrogen production [52]. To gain sufficient light absorption and promote charge transfer from Ru-complex to semiconductor, modification of ligands are required to improve the hydrogen production rate. Tris(2,2′-bipyridine)ruthenium sensitizer (Ru(bpy)_3_), tris(2,2′-bipyridine-4,4′-dicarboxylic acid)ruthenium(II) sensitizer (Ru(dcbpy)_3_) and tris(bipirimidine)ruthenium(II) sensitizer (Ru(bpym)_3_) have been loaded on Pt/TiO_2_ to evaluate the hydrogen production rate. The yield of hydrogen gas by irradiating with a xenon (Xe) lamp (500 W) with 0.1 M ethylenediaminetetraacetic acid (EDTA) as electron donor reagents was 23.3 μL/h with 1.00 mM Ru(bpy)_3_^2+^, and a similar value of 17.8 μL/h was obtained with 0.25 mM Ru(dcbpy)_3_^4−^. The chemical structure of Ru(bpym)_3_^2+^ contains more nitrogen, which showed a higher affinity to the TiO_2_ surface than Ru(bpy)_3_^2+^ and thus resulted in enhanced charge transfer. The results also indicated that the bipyrimidine structure showed the highest hydrogen production rate of 80.1 μL/h [53]. The electron injection rate from Ru(bpy)_2_(dcbpy)^2+^ to TiO_2_ was estimated to be in the range 1.0–5.5 × 10^8^ s^-l^ [54]. The effects of the anchoring groups (carboxylate and phosphoric acid) of Ru-complexes on photocatalytic hydrogen production were evaluated by Choi et al. in detail [55,56]. The yields of hydrogen gas after 3 hours with various numbers of anchoring groups were in the order: P2 (ca. 130 μmol) > P6 (ca. 110 μmol) > C6 (ca. 30 μmol) > C2 (ca. 12 μmol) > (C4 ca. 10 μmol), suggesting that the phosphoric acid group showed a higher hydrogen production capacity (Figure 4(a)) [55, 56].

The adsorption mechanism of bis(tetrabutylammonium) cis-bis(thiocyanato)bis(2,2′-bypiridine-4,4′-dicarboxylato)ruthenium(II) (N719) on TiO_2_ was investigated by Lee et al. [57]. Fourier transform infrared spectroscopy and Raman spectroscopy analyses of the anchoring mode of N719 at the TiO_2_ interface showed that two carboxylic acid groups were anchored on the TiO_2_ surface at a TiOH group, and the carboxylic group exhibited electrostatic bonding (H-bonding) and covalent bonding with a bidentate bridging mode on TiOH groups [57].

#### 4.1.2. Effects of ligand structure of Ru-complex dyes on hydrogen production

Ligands also influence hydrogen production capacity. Maeda et al. investigated a variety of nanosheets that were sensitized by different Ru(II) complexes [58]. The dye-sensitized layered perovskite niobate (KCa_2_Nb_3_O_10_) nanosheet showed effective hydrogen production with turnover numbers (TONs) of 1550, 960 and 12 for Ru−CH_3_, Ru-H and Ru−CF_3_, respectively, under visible-light irradiation. The reason for this result came from the differences between the oxidation potentials of the metal to ligand charge transfer (MLCT) excited states of the Ru(II) complexes (Eox*) and the CB of nanosheets. The MLCT excited state potentials were −1.18 V, −1.08 V and −0.67 V vs Ag/AgNO_3_ for Ru-CH_3_, Ru-H and Ru-CF_3_, respectively, and that of the reference HCa_2_Nb_3_O_10_ nanosheet was −1.13 V vs silver/silver nitrate (Ag/AgNO_3_). The energy potential (LUMO) of Ru-CF_3_ was too negative for the CB of the nanosheet, suggesting that ligands have a strong effect on the LUMO’s energy potential and hydrogen production rate (Figure 4(b)) [58]. Other Ru-complexes have also been investigated. Zheng et al. [59] investigated a 2-hydroxyl-5-(imidazo-[4,5-f]-1,10-phenanthrolin) benzoic acid linked ruthenium-complex. This system showed a hydrogen production rate of 2578 μmol/h under optimal conditions.

#### 4.1.3. Di-nuclear Ru-complex system for hydrogen production

Multi-nuclear Ru-complex systems are also effective for hydrogen production. The design of multi-nuclear Ru-complexes was suggested by Amadelli et al [60]. Multi-nuclear complexes can increase the electron injection rate by adopting an antenna-sensitizer molecular device structure. Based on this system, Peng et al. investigated a binuclear Ru-complex system for photocatalytic hydrogen production [61–64]. The Pt-loaded TiO_2_ showed effective hydrogen production with TONs of 874.3, 561.3 and 271.5 for Ru_2_(bpy)_4_L_1_–PF_6_ (L = μ-4,4-azo-benzene carboxylic acid), Ru(bpy)_2_(him)_2_-NO_3_ (him = imidazole) and N719, respectively, under visible-light irradiation. This result suggested that the bi-nuclear Ru-complex acted as an ‘antenna’ to improve photo-excited charge transfer from the dye into TiO_2_, while preventing backward charge transfer from TiO_2_ to the dye because the electron donor part of the Ru-complex is far away from TiO_2_ [61,62]. A di-Ru-complex, [Ru_2_(bpy)_4_(BL)](ClO_4_)_2_ (BL = bridging ligand), demonstrated efficient hydrogen production with a TON of 2364 and an apparent quantum efficiency (AQY) of 16.8%. On the other hand, when the ligand of [Ru_2_(bpy)_4_(BL)](ClO_4_)_2_ had a carboxylic acid group, i.e. [Ru_2_(dcbpy)_4_(BL)](ClO_4_)_2_, a TON of 317 and an AQY of 1.7% were obtained. Experiments with N719 under the same conditions showed similar performance with a TON of 306 and an AQY of 1.6%. During the photocatalytic reaction, the photo-generated electrons were injected from ruthenium complexes to TiO_2_. The injected electrons can be used for hydrogen production, and they can also be involved in the backward electron reaction from the TiO_2_ surface to the Ru-complex through carboxylic acids such as in [Ru_2_(dcbpy)_4_(BL)](ClO_4_)_2_ and N719. In the case of [Ru_2_(bpy)_4_(BL)](ClO_4_)_2_, the coordination structure between Ru-complex and TiO_2_ was varied as the valence mode of Ru^II^ was changed after electron injection into TiO_2_. Therefore, the backward electron reaction and/or charge recombination in [Ru_2_(bpy)_4_(BL)](ClO_4_)_2_ could be reduced. As a result, the electron injection and hydrogen production efficiencies were increased (Figure 4(c)) [63]. The yield of hydrogen for [Ru_2_(bpy)_4_(BL)](ClO_4_)_2_ (1420 μmol) was higher than that for mono-nuclear Ru-complex [Ru(bpy)_2_(L)](ClO_4_)_2_ (803 μmol), suggesting the antenna effects could enhance the hydrogen production rate [64].

### 4.2. Other metal complexes

**Figure 5.**
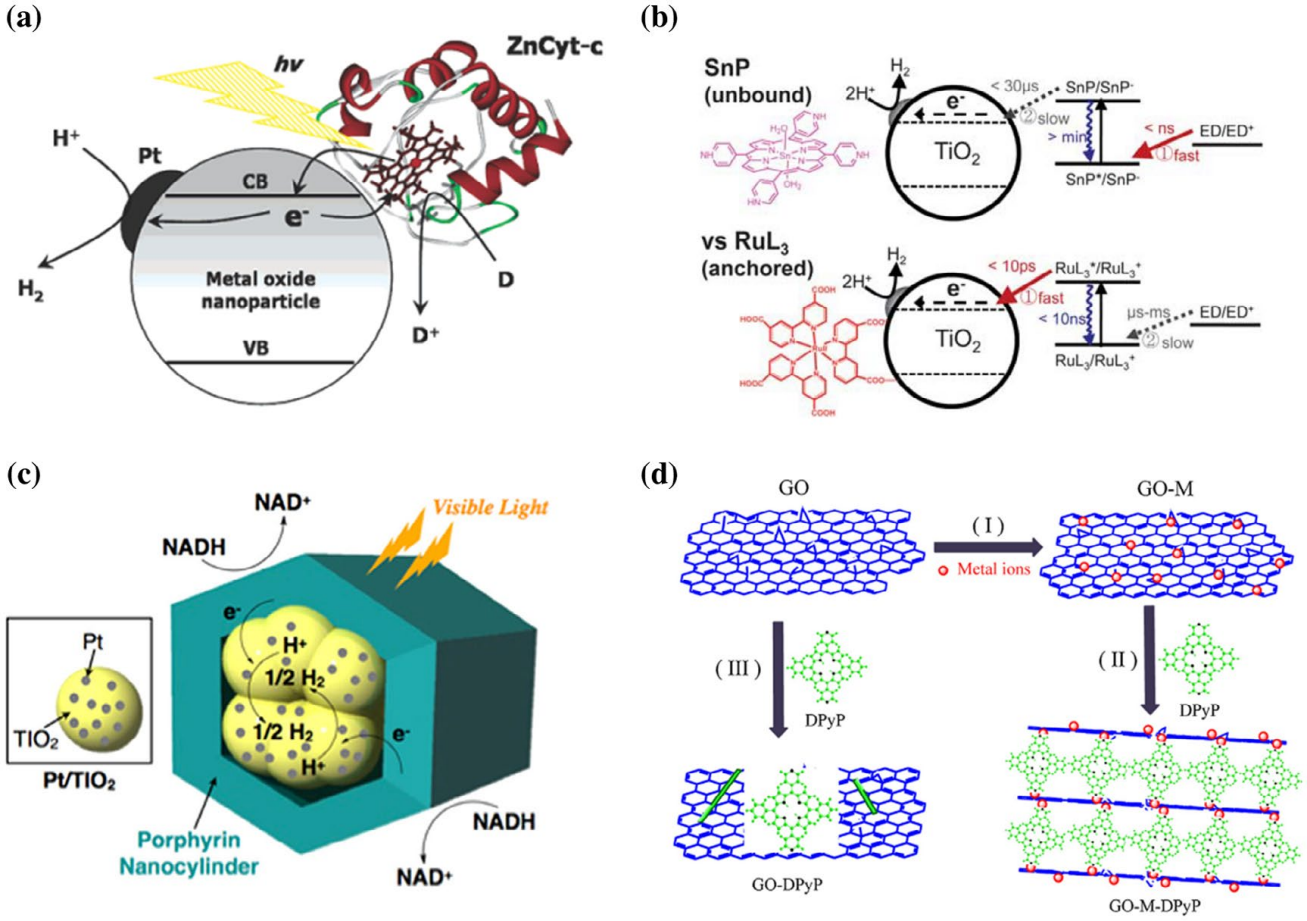
Porphyrin-related organic–inorganic composite systems for photocatalytic hydrogen production. (a) Zinc-treated Cyt-*c* enzyme-sensitized photocatalyst. Reprinted with permission from Ref. [68]. Copyright 2005 American Chemical Society. (b) Sn-porphyrin–TiO_2_ hybrid system. Reprinted from Ref. [69] with permission from The Royal Society of Chemistry. (c) Porphyrin nanocylinder-packed TiO_2_ system. NADH: nicotinamide adenine dinucleotide. Reprinted with permission from Ref. [71]. Copyright 2013 American Chemical Society. (d) Porphyrin–graphene composite photocatalyst. Reprinted with permission from Ref. [73]. Copyright Elsevier.

Ruthenium dyes can use a long charge lifetime associated with a triplet state for hydrogen production, but on the other hand, weak light absorption is a disadvantage [65]. The porphyrin skeleton which is also found in photosynthetic systems has a high light absorption efficiency and a wide light absorption band in the ultraviolet–visible light region, and thus, we will review dye-sensitized photocatalysts utilizing this property. Malinka et al. [66] investigated zinc porphyrin-coated TiO_2_ for photocatalytic hydrogen production, and the catalyst showed a visible range absorbance up to 700 nm. The tris(N-methyl-4-pyridyl)-substituted zinc porphyrin (ZnP^3+^) with 0.4 wt% Pt-loaded TiO_2_ particles and EDTA showed efficient photocatalytic hydrogen production with a TON of 182. The rate of electron injection into TiO_2_ for zinc tetra-carboxyphenyl porphyrin is on the order of femtoseconds (k_inj_ ~ 10^12^ s^−1^), whereas for zinc cytochrome *c* (Zn-Cyt-*c*) enzyme, the electron injection rate was three to four orders of magnitude slower. This efficient electron injection from the triplet state of Zn Cyt-*c* into TiO_2_ electrodes can be used for effective hydrogen production, which was estimated to have a quantum efficiency of 10 ± 5% per absorbed photon (Figure 5(a)) [67,68].

If the porphyrin itself has a long charge lifetime, efficient photoreaction can occur without loading it to a photocatalyst. Kim et al. [69] investigated tin porphyrin (SnP)-sensitized photocatalytic hydrogen production in a TiO_2_ with EDTA electron donor system. The SnP could not be adsorbed on TiO_2_ as a physical/chemical driving force in the solution process. However, effective hydrogen production (TON of 440, AQY = 35% at 550 nm) was found during photoreaction. A photo-induced long-lived radical anion species (SnP^•-^) was generated by a coupled electron donor and had a lifetime long enough to react with the TiO_2_ surface from the bulk solution under photo-irradiation (Figure 5b) [69].

Zhu et al. [70] reported a catalyst with porphyrin directly anchored on platinum nanoparticles. The catalyst comprised 2.2 nm platinum particles, porphyrin dye and weakly bound pyrene via hydrogen bonding with porphyrin. The catalyst raised the porphyrin to the excited state using energy transferred by charge transfer from the absorption of pyrene. Electrons were transferred from the porphyrin to platinum, and hydrogen was produced. The TON and quantum yields of hydrogen were 6311 and 2.65%, respectively.

Hasobe et al. [71] reported a hybrid photocatalyst with a self-assembled cylinder-like structure comprising porphyrin cage and TiO_2_ particles (Figure 5(c)). The nanocylinder structure enhanced charge separation and, thus, drastic enhancement in the hydrogen production rate was observed. The hydrogen production rate for Pt/TiO_2_−ZnP(Py)_4_ nanocylinders was ∼60 mol/g Pt, whereas that for an unencapsulated system comprising Pt/TiO_2_ and ZnP(Py)_4_/Pt nanocylinders was ∼0.3 mol/g Pt.

Zhu et al. [72] reported a 5,10,15,20-tetrakis(4-(hydroxyl)phenyl) porphyrin (TPPH)-adsorbed reduced graphene oxide (RGO) hybrid catalyst. The porphyrin was non-covalently adsorbed on the RGO. The TPPH/RGO/Pt catalyst showed an effective photocatalytic activity with a hydrogen production rate of 5.29 mmol/g and an AQY of 1.7%; the hydrogen production rate with P25-TiO_2_ was 1.52 mmol/g. Aggregation of TPPH/RGO/Pt occurred during photosynthesis, which caused charge quenching. The addition of a surfactant, cetyltrimethylammonium bromide (CTAB), further improved the stability and catalytic hydrogen production rate owing to prevention of TPPH/RGO/Pt aggregation, and a hydrogen production rate of 11.2 mmol/g and an AQY of 3.6% were obtained.

Li et al. [73] investigated chromium (Cr)-loaded reduced graphene oxide (RGO) with 5,15-diphenyl-10,20-di(4-pyridyl)porphyrin (DPyP). The pyridine group of DPyP can be coordinated to the Cr cluster, and thus, DPyP-Cr-RGO with a sandwich-like layered structure was fabricated. Without adding other co-catalysts, RGO, DPyP-RGO and DPyP-Cr-RGO showed visible-light-driven hydrogen production, with yields of 137, 686 and 928 μmol/g, respectively (Figure 5(d)).

Yuan et al. and Li et al. evaluated other porphyrin sensitizers and composites. Yuan et al. [74] reported tetrakis(4-carboxyphenyl)porphyrin dye-sensitized molybdenum sulfide (MoS_2_)/ZnO. This system showed a TON of 516 at > 420 nm. Li et al. [75] reported a 5,10,15,20-tetraphenylporphyrin (THPP)-coated copper (I) oxide (Cu_2_O) photocatalyst. The Cu_2_O/THPP photocatalyst yielded a 5% photocatalytic activity, evolving 1.3 mmol/g after 6 hours, which suggested that high dye-sensitized photocatalytic activity was achieved with a Cu_2_O system.

## 5. Metal-free dye-sensitized hydrogen production

In the case of metal-free organic dye-sensitized photocatalysts, charge injection is inefficient due to easily occurring charge recombination or backward electron reaction. For coumarin pigments, the addition of 4-tert-butylpyridine (TBP) is necessary to prevent charge recombination in order to demonstrate performance equivalent to Ru-dye [76]. This result indicates that the charge recombination lifetime of metal-free dyes is shorter than that of Ru-dyes, which will reduce the hydrogen production rate. To increase the hydrogen production rate when using metal-free organic dyes, precise molecular design is required to ensure efficient charge transfer. To investigate this system, covalently adsorbed metal-free dyes on metal oxides such TiO_2_ were investigated. Houlding and Grätzel [77] reported hydrogen generation under visible light using an 8-hydroxy-orthoquinoline metal-free dye with Pt-loaded anatase TiO_2_. The hydrogen production rate was as high as 750–1000 μL/h, and the TON was over 40. Other organic dyes also showed dye-sensitized photocatalytic properties such as xanthene dyes [78−81]. Eosin Y demonstrated a high hydrogen production rate for the xanthene dyes. A variety of Eosin Y dye-sensitized TiO_2_ photocatalysts were investigated with various TiO_2_ systems, such as rhodium (Rh)-TiO_2_ [82], copper(II) oxide (CuO)/TiO_2_ [83], mesoporous TiO_2_ [84], TiO_2_–SiO_2_ mixed oxide [85] and aeroxide TiO_2_ [86]. Among these combinations, aeroxide TiO_2_ showed the highest hydrogen production rate with a 12.2% AQY by optimization of irradiation energy and intensity.

Electron donors such as EDTA, triethanolamine (TEOA), ascorbic acid, oxalic acid and alcohol have been investigated. The concentration and pH were shown to strongly affect the hydrogen production rate [66].

Coumarin and merocyanine derivatives could also be used for effective visible-light-driven photocatalysts. A merocyanine dye together with an I_2_ sacrificial reagent showed high photocatalytic activity [87]. This system showed a higher hydrogen production rate than an EDTA system. External quantum efficiencies (EQYs) of 1.8% and 2.5% were obtained with NK-2045 (merocyanine dye) at 517 nm and C-343 (Coumarin) at 440 nm, respectively [88].

**Figure 6.**
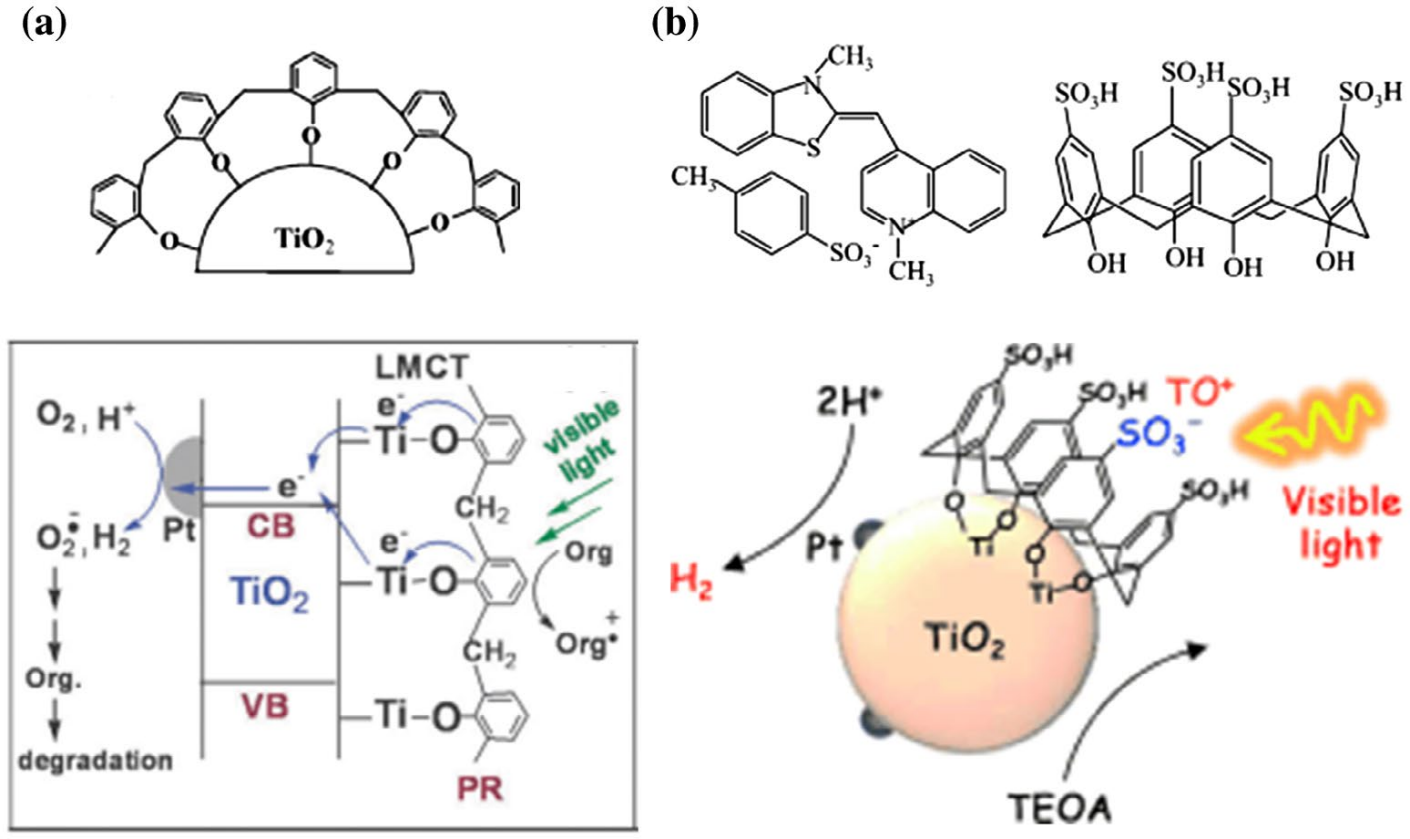
Phenol–anchored polymer/oligomer structure and TiO_2_ composite systems for photocatalytic hydrogen production. (a) A hybrid system of TiO_2_ and phenolic resin acting as a visible-light sensitizer. LMCT: ligand to metal charge transfer. PR: phenolic resin. Reprinted from Ref. [90] with permission from The Royal Society of Chemistry. (b) Photocatalyst–calixarene–cationic dye three-component system. Reprinted with permission from Ref. [91]. Copyright 2013 John Wiley & Sons.

Ikeda et al. [89] reported benzyl alcohol (Bn(OH)_2_)-type photocatalysts for visible-light-driven photocatalytic hydrogen production. The phenol moieties of alcohol groups were chemically bonded to a TiO_2_ surface and showed hydrogen production under visible-light irradiation, which indicated effective charge transfer. Zhang and Choi [90] investigated phenolic resin as a VL sensitizer. The resin can be coated successively on TiO_2_, and it showed a new absorption band up to 650 nm (Figure 6(a)). This ligand-to-metal charge transfer showed effective hydrogen production. Kamegawa et al. [91] investigated calixarenes as a sensitizer. This sensitizer has a sulfonic group on the phenol moiety, the negative charge of which could accept the cationic thiazole orange. The photocatalyst–calixarene–cationic dye three-component system broadened the absorption spectra and showed effective charge transfer from the cationic dye to the photocatalyst through calixarene. The AQY was up to 10.4% at 460 nm (Figure 6(b)).

To increase the hydrogen production rate of metal-free dye-sensitized photocatalysts, the design of organic dyes can play a critical role in improving charge extraction during photosynthesis. In metal-free dye-sensitized solar cells, sensitizers of a donor–bridge–acceptor-type were used [92]. The donor–acceptor structure could separate charge upon photoexcitation. The bridge moiety could increase light absorption over a wide wavelength region due to reduction of the HOMO–LUMO band gap and increase in the charge separation lifetime. The donor part acts as a light harvesting moiety, which determines the light harvesting efficiency of the dye. Choi et al. [93] investigated a triphenyl amine (D)–thiophene (B)–acrylic acid (A) system for visible-light-driven photocatalytic reactions. The multiple anchoring groups could increase the extinction coefficient of the CT transition state due to an increase in the donor–acceptor interaction between π-chromophores. This system showed an increase in the hydrogen production rate with an increasing number of anchoring groups. The multi-anchoring groups also showed better stability during hydrogen production. This result indicated that multi-anchoring groups could improve the hydrogen production rate.

**Figure 7.**
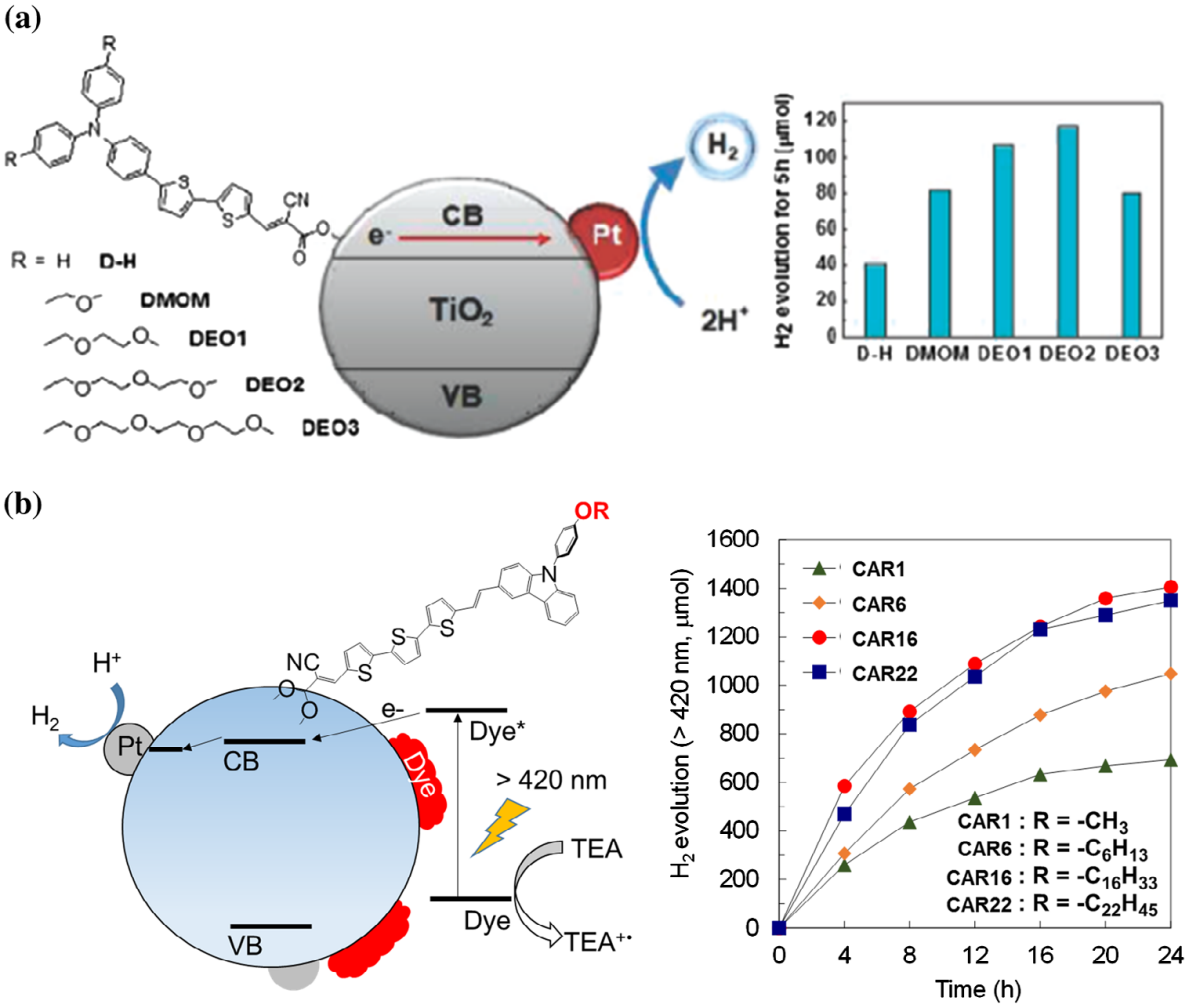
(a) Hydrophilic triphenylamine-thiophene–TiO_2_ hybrid system. Reprinted with permission from Ref. [94]. Copyright 2010 American Chemical Society. (b) Hydrophobic carbazole-thiophene–TiO_2_ hybrid system. Reprinted from Ref. [96] with permission from The Royal Society of Chemistry.

Lee et al. [94] investigated the hydrophilic effects using a triphenyl amine (D)–dithiophene (B)–acrylic acid (A) system (Figure 7(a)). When the impact of hydrophilicity was increased by changing the number of ethylene glycol moieties, the hydrogen production was improved by as much as three times. The AQY of this system at 426 nm was 0.27% [95]. The water molecules and hydrophilic groups interacted at the excited state, which led to a slow charge recombination rate. This slow charge recombination improves the hydrogen production. On the other hand, hydrophobic groups also affect the hydrogen production rate. This phenomenon has been reported by Lee group and our group [96,97]. We investigated the effect of the number of alkoxy chains that was varied in a carbazole (D)–terthiophene (B)–carboxyacrylic acid (A) moiety. Introducing hydrophobic effects also improved the hydrogen production rate by two times compared with bare conditions. The highest TON value with dye CAR16 was 3094 after 24 hours, whereas a TON of 1497 was obtained under bare conditions (CAR1). The differences arose from the interface between the dye and the water medium. The diameters of the largest semicircle in the impedance spectra decreased in the order CAR16 (293.4 Ω) > CAR22 (249.3 Ω) > CAR6 (222.7 Ω) > CAR1 (205.2 Ω), suggesting that the interface between the organic layer and the water medium trapped electrons when the hydrophobicity decreased. These results also indicated that the alkoxy group impacted the hydrogen production (Figure 7(b)) [96]. Lee et al. [97], Manfredi et al. [98] and Tiwari et al. [99] also reported similar phenomena. Manfredi et al. [98] compared the effects of hydrophilic groups and hydrophobic groups on hydrogen production, and the results showed higher hydrogen production rate with hydrophobic groups (PTZ-ALK dye: 1060 μmol after 20 hours) than with hydrophilic groups (PTZ-TEG dye: 421 μmol after 20 hours).

The hydrogen production rate was lower than that in our case, probably because the bridge group reduced the charge recombination rate. To solve this issue, we have systematically investigated a phenothiadine (D)–oligothiophene (B)–aclilcarboxilic acid (A) system. The system without oligothiophene showed a TON of only 483 after 16 hours and an EQY of 0.03% at 420 nm [97].

**Figure 8.**
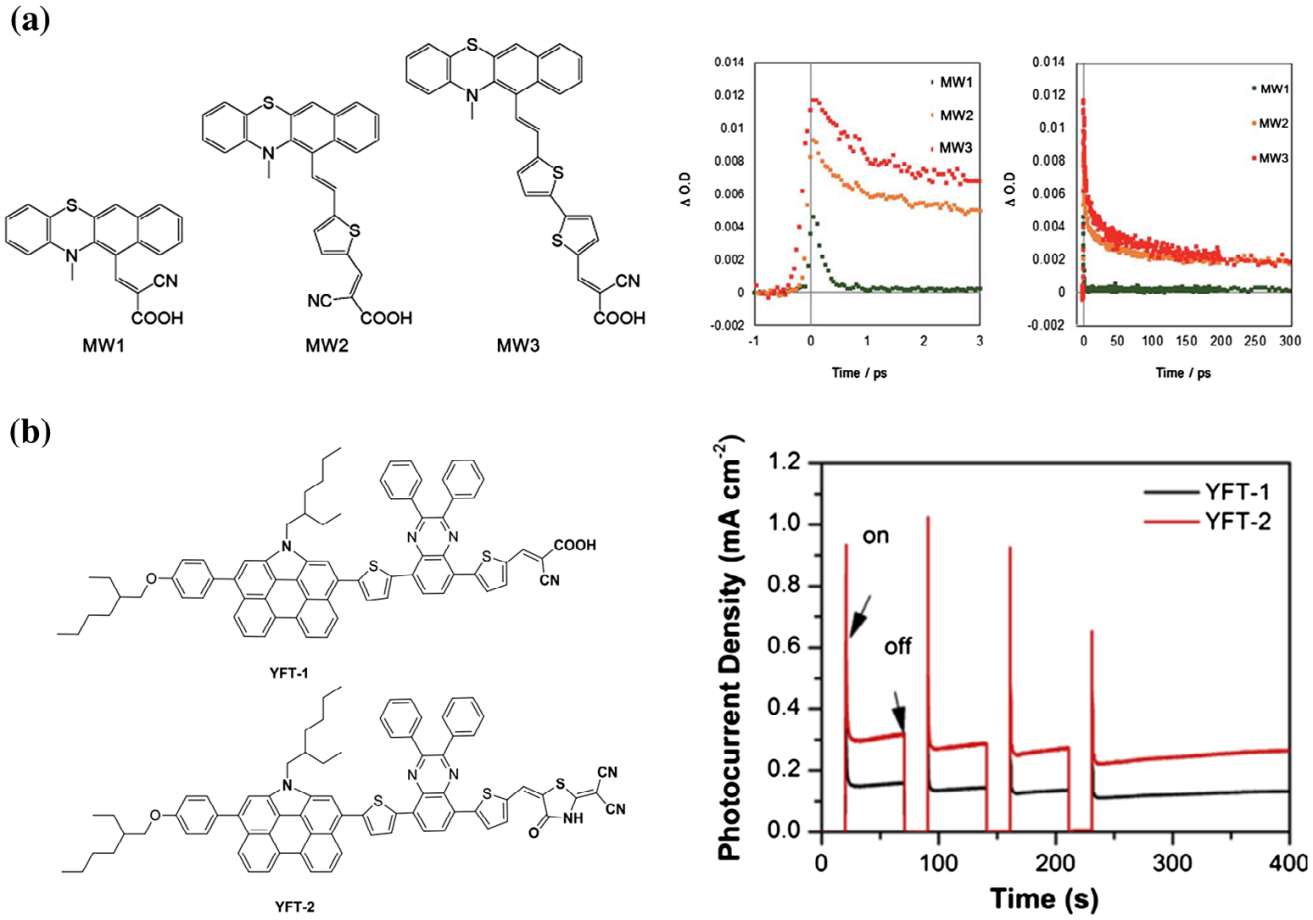
Bridge and anchor effects on dye-sensitized photocatalytic hydrogen production. (a) Phenothiazine-thiophene (MW1–MW3)–TiO_2_ hybrid systems, and their transient absorption decay profiles. Reprinted from Ref. [100] with permission from The Royal Society of Chemistry. (b) Cyanoacrylic acid (YFT-1) and triazole (YFT-2) as anchoring groups and the corresponding photocurrent decay profiles. Reprinted with permission from Ref. [103]. Copyright Elsevier.

However, the hydrogen production rate was dramatically increased when a thiophene bridge was inserted between the donor and the acceptor. A TON as high as 4460 and an EQY of 1.65% at 420 nm were obtained, which were almost nine times higher than those without the thiophene bridge group. These results indicated that the bridge group increased the number of extracted electrons in photosynthesis. The transient absorption spectra of these photocatalysts showed different charge recombination rates. The MW1 dye showed a shorter decay charge recombination component of τ1 (0.18 ps), suggesting the charge was shortly quenched after electron injection into the TiO_2_. On the other hand, when the thiophene rings were inserted in the sensitizer, longer components in the decays of MW2 (47.0 ps) and MW3 (85.4 ps) were found. This fact suggested that the thiophene bridge slowed down recombination between the dye and the TiO_2._ Therefore, MW1 showed a lower TON and quantum efficiency. The longer decay components can provide long-lived electrons in TiO_2_, and thus the rate of hydrogen production can be increased (Figure 8(a)) [100].

Pal et al. [101] have developed DBA-based dyes using triphenylamine as a donor.When a hydrophobic group such as butoxy group was used for triphenylamine derivatives, hydrogen production capacity was improved. Furthermore, when cyclopentadithiophene derivatives were used as bridges, the performance was improved.

Narayanaswamy et al. [102] developed dyes that used dithiafulvalenes as a donor and reported that a TON of up to 9664 was obtained. Yu et al. [103] investigated the influence of anchoring groups by using cyanoacrylic acid (YFT-1) and triazole (YFT-2). The dye-sensitized solar cell performance was 7.58% for YFT-1 and 4.43% for YFT-2, and the hydrogen production rate was 62.3 μmol/h for YFT-1 and 83.5 μmol/h for YFT-2. YFT-2/TiO_2_ showed a higher photocurrent value than YFT-1/TiO_2_ in a TEOA solution, suggesting efficient charge transfer in the triazole system (Figure 8(b)). Thus, with new substituents, the reactivity of molecules can change significantly even with metal-free dyes. These results mean that more efficient dye-sensitized photocatalysts can be developed by designing new dyes.

## 6. Organic semiconductor-type photocatalysts

Carbon materials have attracted much attention for use in photocatalytic hydrogen production because the carbon source is abundant on earth. Carbon nitride (C_3_N_4_) with a carbon-nitride polymeric structure showed a small band gap of less than 3.0 eV and effective photocatalytic activity in water medium for hydrogen production [20]. Pt or RuO_2_ catalysts can be supported by C_3_N_4_, which improved the catalytic activity for water splitting due to the co-catalytic activity for H_2_ or O_2_ formation [104].

Due to the wide band gap (ca. 2.7 eV), C_3_N_4_ does not absorb light above 600 nm. To overcoming this issue, band-gap engineering of C_3_N_4_ was performed to tune the band gap of C_3_N_4_ in a wide range (400–700 nm) and change the thermal polymerization temperature from precursors such as melanin, cyanamide, dicyanamide, urea and thiourea [20, 105]. A method for improving the hydrogen production rate by coexistence with a dye and C_3_N_4_ was reported by Takanabe et al [106]. They used phthalocyanato-magnesium (PcMg) as an organic sensitizer, which was shown to absorb light from 400 to 1100 nm. The dye-sensitized-type photocatalytic hydrogen production was demonstrated using this system, which showed an AQY of 0.07% at 660 nm with TEOA as sacrificial reagents. Yu et al. [107] and Zhang et al. [108] used Zn-Pc as a sensitizer, with carboxylic acids as the anchoring group. This sensitizer showed a sharp peak at 700 nm in solution, and a similar absorption spectrum was obtained after it was anchored to C_3_N_4_ powder, suggesting that the sensitizer self-assembled on C_3_N_4_. The dye-loaded C_3_N_4_ showed effective hydrogen production with an AQY of as high as 1.85% at 700 nm. Min and Lu [109] reported a variety of organic sensitizers with C_3_N_4_ of photocatalytic activity as 19.4% at 550 nm. Compared with Rose Bengal, fluorescein, Rhodamine B and Ru(bpy)_3_Cl_2_, the Eosin Y showed the highest photocatalytic activity. Yan and Huang [110] reported polymer-sensitized C_3_N_4_. Polythiophene (P3HT) is a well-known photo-responsive semiconducting material. P3HT-coated C_3_N_4_ showed a 300 times increase in the hydrogen production rate when 3 wt% of P3HT was used, and an AQY of 2.9% at 420 nm was obtained with Na_2_S sacrificial reagents, suggesting effective charge transport during the photoreaction. Ascorbic acid showed a smoother electron transfer pathway than Na_2_S and, therefore, it showed a remarkably higher hydrogen production rate with an EQY as high as 77.4% at 420 nm [111].

Chen et al. [112] reported a photocatalyst based on a graphitized polyacrylonitrile (g-PAN)/C_3_N_4_ composite, where the graphitized polyacrylonitrile was directly grown on the layered C_3_N_4_. This composite showed good charge separation and transfer between g-PAN and C_3_N_4_, and thus efficient hydrogen evolution was achieved; the hydrogen production rate was 37 μmol/h.

The photocatalytic activity of C_3_N_4_ can also be enhanced by combining with inorganic photocatalysts. Li et al. [113] reported a composite of TaON and C_3_N_4_, which showed that electron transfer from the CB of C_3_N_4_ (−1.12 eV vs SHE) to the CB of TaON (−0.34 eV vs SHE) occurred during VL photoexcitation. Photodegradation of rhodamine B by TaON-C_3_N_4_ composite was demonstrated under visible-light irradiation. Although this system was not used as a photocatalyst for hydrogen production, it showed the possibility of charge separation between C_3_N_4_ and semiconductor materials.

**Figure 9.**
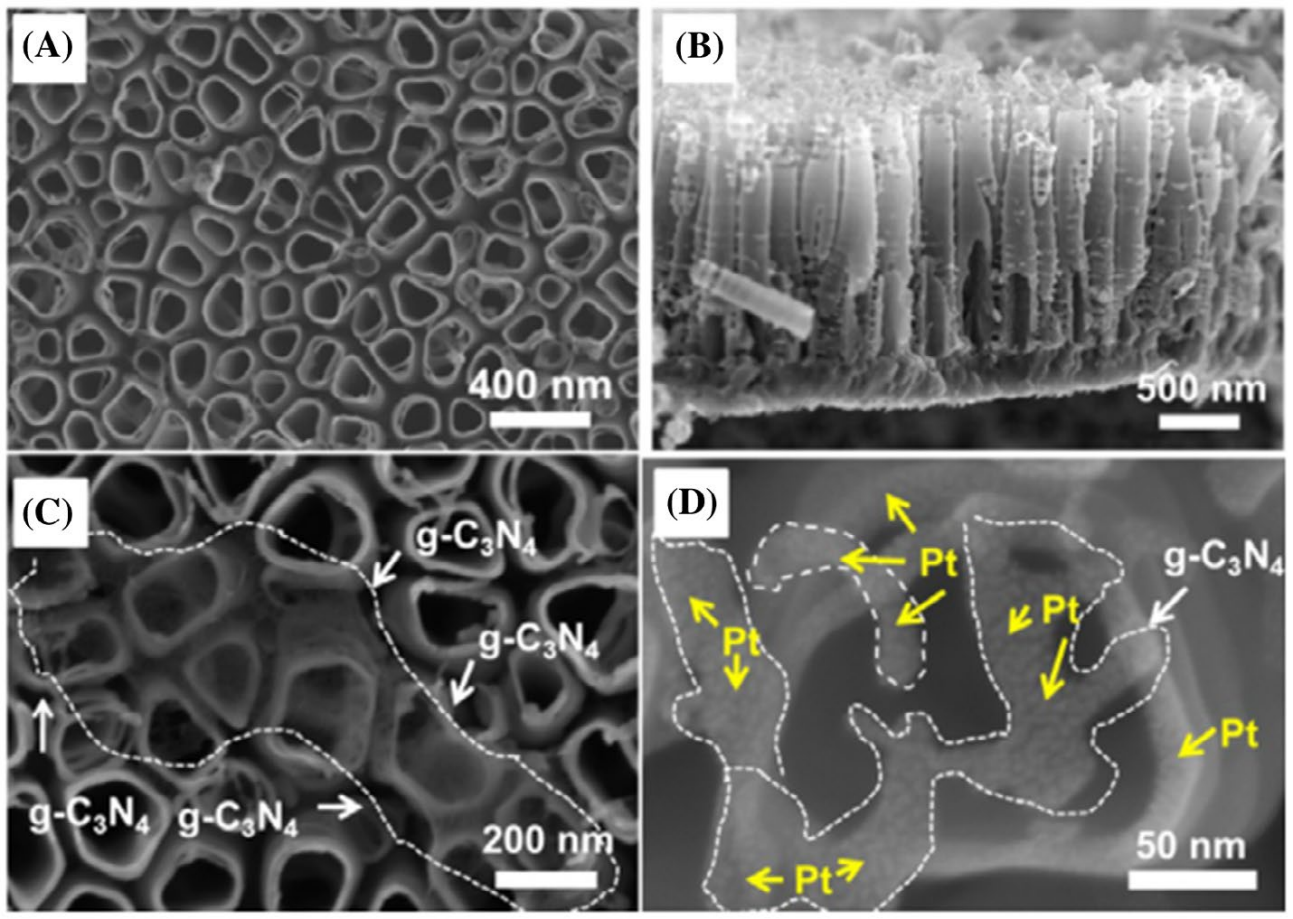
Scanning electron microscopy images showing (a) top and (b) side view of TiO_2_ nanotube arrays, (c) TiO_2_ nanotube/C_3_N_4_ and (d) TiO_2_ nanotube/C_3_N_4_/Pt. Reprinted with permission from Ref. [115]. Copyright 2016 John Wiley & Sons.

Yan and Yang [114] reported a C_3_N_4_/TiO_2_ photocatalyst. Reasonably fast electron transfer from the CB of C_3_N_4_ (−1.12 eV vs SHE) to the CB of TiO_2_ (−0.29 eV vs SHE) occurred during VL photoexcitation. The particle size of TiO_2_–50 wt% C_3_N_4_ composite materials was estimated to be approximately in the range 30–40 nm. The composite structure can efficiently separate electron–hole pairs between TiO_2_ and C_3_N_4_; therefore, a hydrogen production rate as high as 22.5 μmol/h was obtained. The structure of semiconductors also affects charge extraction in photosynthesis. TiO_2_ nanotubes facilitated charge separation in the orthogonal array shown in Figure 9. The combination of C_3_N_4_ and TiO_2_ nanotubes increased the hydrogen production rate to 15.62 μL/h cm^2^, whereas the hydrogen production rate for the device containing only TiO_2_ nanotubes was 0.16 μL/h cm^2^ (Figure 9) [115].

## 7. Co-catalysts in organic–inorganic composites

Co-catalysts are important for improving the water-splitting activity of organic–inorganic composites. The co-catalyst serves as a reaction site that catalyzes the reaction. In addition, it promotes efficient charge separation between co-catalyst and catalyst to achieve efficient hydrogen production [116]. Platinum is commonly used for photocatalytic hydrogen production due to its low overpotential and excellent kinetics. However, platinum is an expensive and rare metal, which will become a bottleneck in the commercialization stage. To overcome this issue, research and development of low-cost catalysts is urgently required. Trasatti [117, 118] reported that the hydrogen production reaction at the electrode depends on the binding energy between the metal surface and hydrogen and that there is a volcanic relationship. Both acidic and basic platinum, rhodium, gold, nickel and ruthenium have shown high co-catalytic ability. A similar tendency was also observed with a powdery co-catalyst, and Pt, Rh, gold (Au), nickel oxide (NiO) and RuO_2_ exhibited high activity [4].

Platinum metal complex-based organic dyes have been investigated for hydrogen production. Pt(alizarin)_2_ dye-loaded TiO_2_ particles showed a higher hydrogen production rate than alcidine yellow-coated Pt-TiO_2_ [119]. Zhang et al. [120] reported a co-catalyst system comprising platinum(II) diimine dithiolate sensitizer-loaded Pt–TiO_2_ particles. The Pt-1 sample showed the highest hydrogen production rate, with a TON of as high as 84, whereas samples 2, 3 and 4 showed TONs of 72, 5 and 7, respectively; samples 3 and 4 were not bound to TiO_2_. Jarosz et al. [121] also investigated a platinum(II) terpyridyl acetylide-type sensitizer with TiO_2_ for photocatalytic hydrogen production. In contrast to the di-imine dithiolate system, terpyridyl acetylides with carboxylic anchoring groups showed a lower hydrogen production of TON = 16 than that without carboxylic anchoring groups (TON = 115), due to oxidative quenching occurring during the reaction.

**Figure 10.**
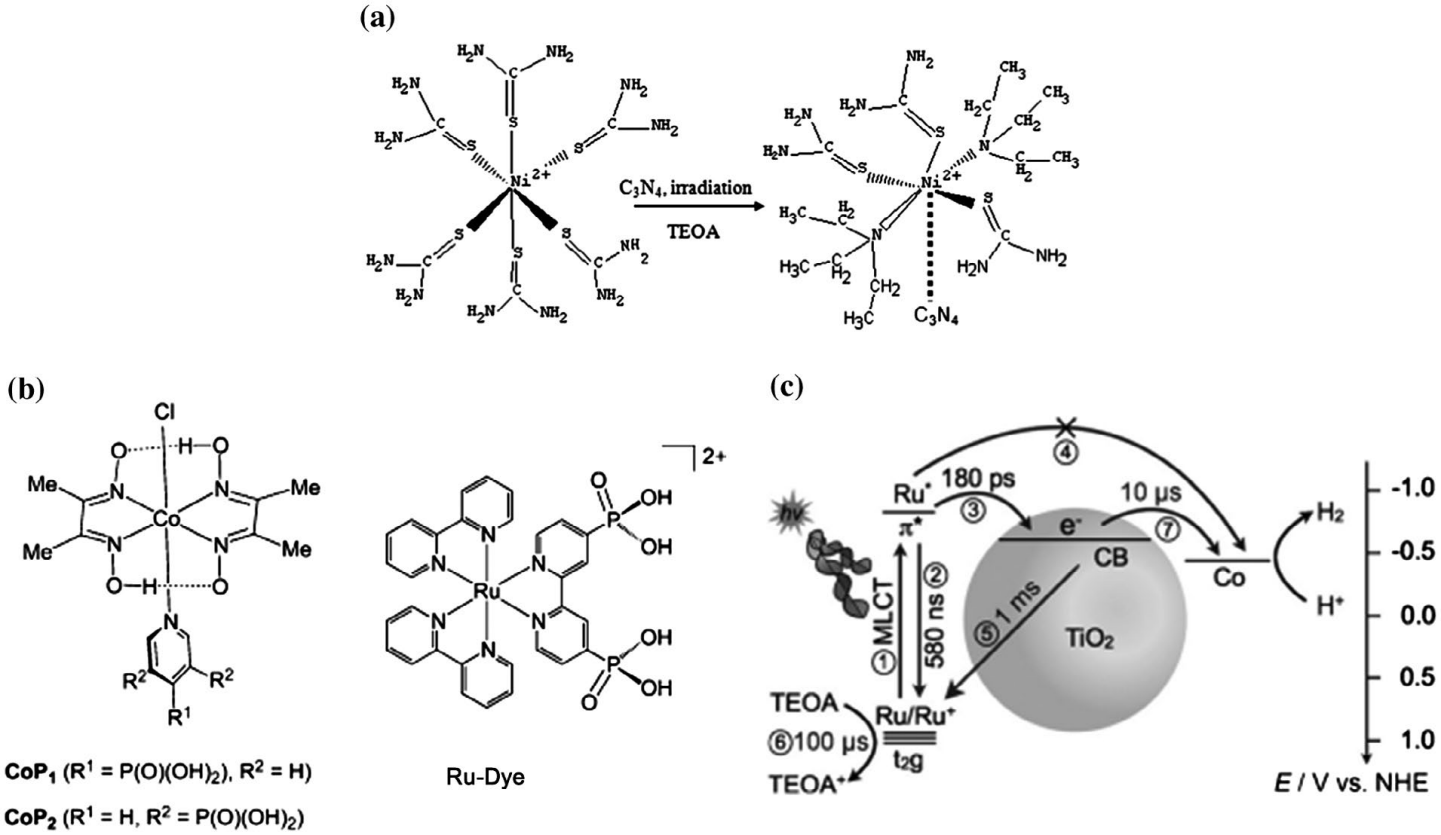
Organic nickel and cobalt complexes as co-catalysts for photocatalytic hydrogen production. (a) Structure of a nickel-complex–TiO_2_ hybrid system. Reprinted from Ref. [125] with permission from The Royal Society of Chemistry. (b, c) Ruthenium-complex sensitizer–TiO_2_–cobalt-complex system for photocatalytic hydrogen production. Reprinted with permission from Ref. [130]. Copyright 2012 John Wiley & Sons.

Among non-noble metals, nickel shows the highest activity. Ni-metal organic complexes have been investigated for use as co-catalysts for hydrogen production. Wilson et al. and Jacobsen et al. investigated a 1,3,5,7-tetraphenyl-1,5-diaza-3,7-diphosphacyclooctane ligand-based Ni-complex [122−124]. This Ni-complex showed electrocatalytic activity with trifluoroacetic acid (TFA) or triethylamine (TEA) as a proton donor, suggesting the nickel (Ni)-complex could be used as a reaction centre for hydrogen production. Chen et al. [125] reported a Ni-thiourea complex for photocatalytic reaction. After the Ni-thiourea complex was mixed with a C_3_N_4_ semiconductor, the excited electrons were transferred from C_3_N_4_ to the Ni-thiourea complex (Figure 10(a)). Das et al. [126] reported a water-soluble ligand-capped CdSe photocatalyst with a Ni-complex dye for co-catalytic hydrogen production. A TON of > 280000 was achieved with ascorbic acid as sacrificial reagent at pH 4.5. Reynal et al. [127] reported dye-sensitized-type photocatalytic hydrogen production with a Ni-complex as co-catalyst. The combination of ruthenium-complex/TiO_2_/Ni-complex system showed that the electrons were transferred from the ruthenium-complex sensitizer to the Ni-complex co-catalyst through TiO_2_ particles. The optimized system showed a TON of 524 after 30 hours.

Organic cobalt (Co)-complexes also showed a catalytic hydrogen production rate [128]. Reisner et al. [129] developed a dimethylglyoxime-ligand-type Co-complex for dye-sensitized-type photocatalytic reaction. The combination of TiO_2_/Co-complex with UV irradiation showed effective hydrogen production at 6.07% (AQY) after 4 hours, which suggested that the Co-complex could be used as a co-catalyst for photocatalytic reactions. The combination of RuP/CoP-TiO_2_ showed visible-light-driven hydrogen production at 2.29% (AQY) at > 420 nm after 8 hours (Figure 10(b)) [130]. The mechanism involved two-electron reductions of cobalt-complexes by TiO_2_ particles that were then used for hydrogen production [131]. In the system comprising Ru-dye/TiO_2_/Co-complex, the electrons were injected into TiO_2_ nanoparticles from the Ru-dye within 180 ps. The electrons in the TiO_2_ then migrate to the Co-complex within 10 μs (Figure 10(c)) [130]. Modifying the ligand of Co-complexes can also improve the hydrogen production rate. For a cobalt complex with a pyridyl ligand, visible-light-driven photocatalytic hydrogen production was achieved with an AQY of 0.35% at 465 nm [132].

## 8. Z-scheme-type photocatalysts

Another type of photocatalyst is the Z-scheme-type photocatalyst, which mimics the process in plant photosynthesis (PSI and PSII). In 1979, a two-step water-splitting system involving excitation of a combination of two semiconductors was proposed by Bard [133]. In this system, the charges are separated between two n-type semiconductors via a mediator, which can suppress recombination. Therefore, the efficiency of water splitting can be increased. The system contains photocatalysts for O_2_ evolution and H_2_ evolution and an electron mediator. The mediator passes the electron on to the H_2_ generation photocatalyst, and then a second charge separation process occurs due to light absorption. After the Z-scheme-type photocatalytic reaction system was developed, several p- and n-type semiconductors were developed for utilizing this reaction mechanism. The p-type semiconductors such as WO_3_, TiO_2_, strontium titanate (SrTiO_3_) and BiVO_4_ can be used for the oxidation reaction to produce O_2_, and the n-type semiconductors such as TiO_2_, SrTiO_3_, TaON, zirconium oxide (ZrO_2_) and C_3_N_4_ are used for the reduction reaction to generate H_2_ [134,135].

**Figure 11.**
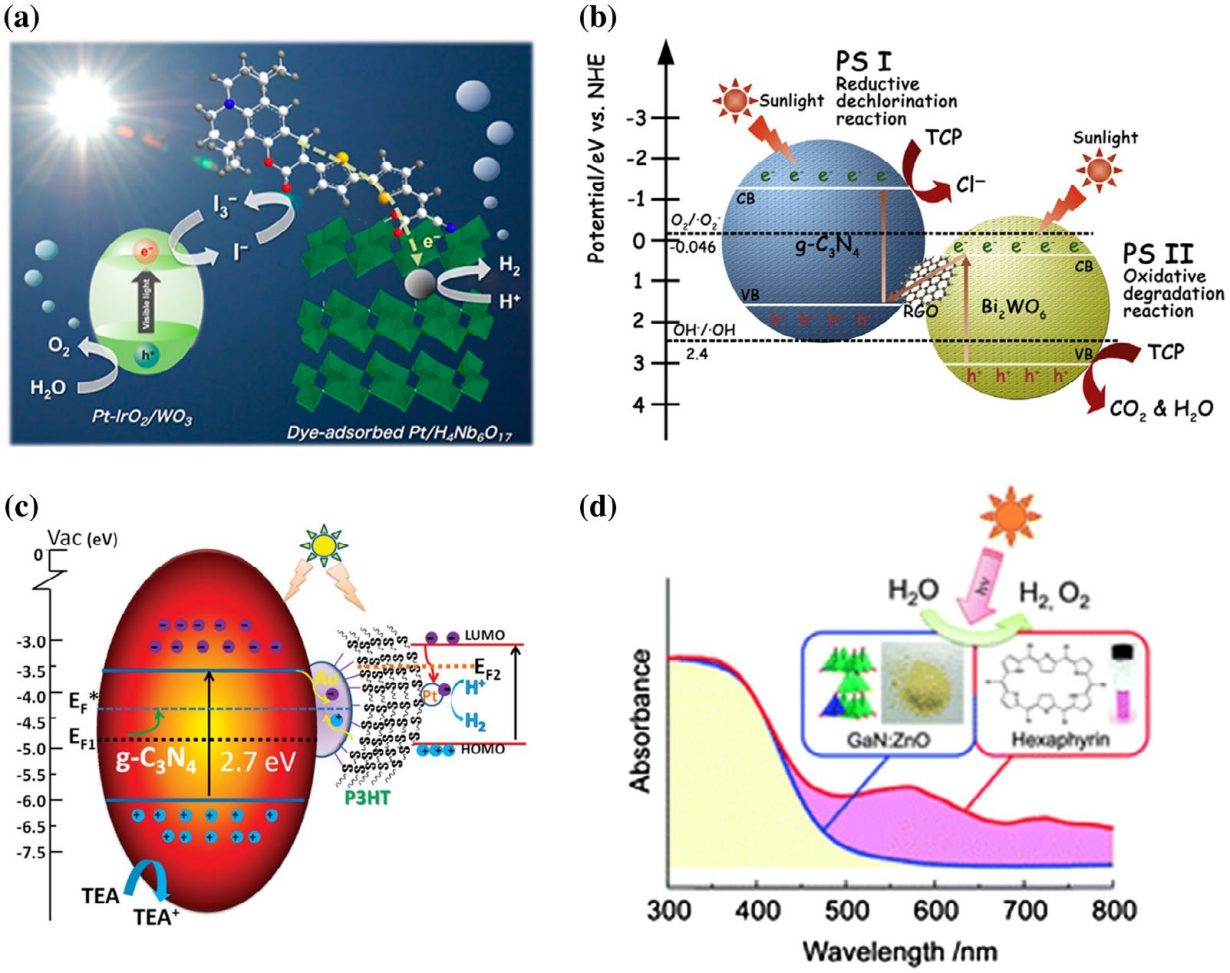
Z-scheme-type organic–inorganic composite system for photocatalytic hydrogen production. (a) Dye-sensitized nanosheet with WO_3_ hybrid system. Reprinted with permission from Ref. [138]. Copyright 2013 American Chemical Society. (b) RGO-mediated C_3_N_4_–BiWO_4_ hybrid Z-scheme-type photocatalyst. Reprinted with permission from Ref. [140]. Copyright Elsevier. (c) Au-mediated C_3_N_4_–P3HT hybrid Z-scheme-type photocatalyst. Reprinted from Ref. [145] with permission from The Royal Society of Chemistry. (d) Hexaphyrin–GaN:ZnO composite photocatalyst. Reprinted from Ref. [153] with permission from The Royal Society of Chemistry.

Grätzel [136] discussed the Z-scheme-type electron transfer in a complete water-splitting system, using a dye-sensitized photocatalyst. This system used a mesoporous WO_3_ film as a catalyst for oxygen production, and a dye-sensitized mesoporous TiO_2_ film as a catalyst for hydrogen generation. The powder-type photocatalytic Z-scheme reaction was reported by Abe et al. [137, 138]. A coumarin-based dye-sensitized Z-scheme water-splitting system, with iridium oxide (IrO_2_)-Pt/WO_3_ and Pt/H_4_Nb_6_O_17_ as photocatalysts and I^-^/I^3-^ as the mediator, showed complete water splitting under VL irradiation. The AQY was 0.05% at 480 nm (Figure 11a).

Solid-state materials can also be used as mediators. A mixture of Ru/SrTiO_3_:Rh and BiVO_4_ particles was co-loaded on RGO, and effective charge transfer occurred from BiVO_4_ to SrTiO_3_:Rh, similar to a Z-scheme reaction [139]. This result indicates that RGO can be used as a mediator for photocatalysts. The composite of C_3_N_4_/RGO/bismuth tungsten oxide (Bi_2_WO_6_) also showed effective electron transfer, although this system did not show hydrogen production (Figure 11(b)) [140]. Similar systems have also been investigated by using combinations of C_3_N_4_/BiVO_4_ [141] and C_3_N_4_/AgPO_4_ [142]. Katsumata et al. [143] investigated a WO_3_/C_3_N_4_ composite, and this composite with TEA as an electron donor was demonstrated for Z-scheme-type hydrogen production (AQY: 0.9% at 405 nm). Martin et al. [144] used Pt-WO_3_ and Pt-C_3_N_4_ with NaI as the redox mediator. This system was capable of complete water splitting, with a H_2_/O_2_ yield of 74/37 μmol/h. An Ag-C_3_N_4_/Au/P3HT/Pt system also showed VL Z-scheme hydrogen production from water, although complete water splitting was not achieved. In this system, C_3_N_4_ and P3HT were separated by gold nanoparticles. Under photo-irradiation, the electrons were transferred from C_3_N_4_ to Au, and the holes in P3HT moved to Au. The electron–hole pairs reacted on the Au due to quenching, and the electrons in P3HT can thus react with protons to produce hydrogen on a platinum co-catalyst (Figure 11(c)) [145].

Organic materials can also be used as mediators. Hagiwara et al. [146−148] reported zirconium (Zr)-doped potassium tantalate (KTaO_3_) modified with an organic molecule, which completely decomposed water via Z-scheme electron transfer. Due to the wide band gap of KTa(Zr)O_3_ (3.0 eV), it cannot absorb visible light without being coated with an organic dye. When the Pt/organic dye/KTa(Zr)O_3_ powder was suspended in pure water and shone with UV-Vis light, both hydrogen and oxygen were produced [147,148]. This result indicated that complete water splitting was achieved. In this system, the UV light was used to excite KTa(Zr)O_3_ and charge was separated between the CB and the VB. The separated electrons were transferred to the organic dye due to lower energy potentials relative to the CB of KTa(Zr)O_3_. The electrons on the organic dye were promoted again by a second excitation. These electrons finally migrated to the surface of the Pt co-catalyst, and hydrogen was then produced. The separated holes were used to produce oxygen.

The decay profile of the photo-induced voltage for KTa(Zr)O_3_ showed a half-life of 11.2 μs. After being treated with organic molecules such as chromate porphyrin (Cr-TCPP), the photo-induced voltage half-life increased to 100 μs. This result suggested that photo-excited charge separation of holes and electrons was enhanced by coating the semiconductors with organic molecules to enable a Z-scheme-type reaction [146].

The organic molecules that can be used include vitamin B12, porphyrin, phthalocyanine, Ru(bpy)_3_, Cu(bpy)_3_, pelerine, coronene and xanthene [149−151]. The LUMO energy of the organic molecules plays an important role in the reaction. After examining many dyes, it is found that when the LUMO energy has a potential balance just between the CB of inorganic oxides and platinum, electrons pumped up by the pigment can efficiently migrate to platinum and the hydrogen production efficiency is maximized [149,150].

Modification of the organic molecule showed a positive effect on the water-splitting activity. Hagiwara et al. [152] estimated the aggregation structure of porphyrins on photocatalysts. The parent Cr-porphyrin-treated KTa(Zr)O_3_ showed characteristic absorption spectra around 613–531 nm for Q bands; however, the alkylated Cr-porphyrin-treated catalysts showed red-shifted and broad peaks around 644–532 nm. These results suggested that the alkylated Cr-porphyrins were J-aggregated and accumulated on the semiconductor. A J-aggregation geometry will not only enhance the absorption range, which can enhance the frequency of excitation, but also enhance charge transport. An increased water-splitting efficiency for hydrogen production was achieved. As a result, the gas generation rate of H_2_/O_2_ was 53.7/29.4 μmol/h, which is higher than that with the parent Cr-TPP (40.0/22.7 μmol/h). To increase the light to energy conversion efficiency, harvesting and utilization of visible light are important. By modifying the structure of the porphyrin, it is possible to adjust the HOMO–LUMO gap by extending the structure of the ring. The absorption spectrum shows a wide absorption band from ultraviolet to the near-infrared region. In addition, the choice of semiconductor also affects the photocatalytic activity. The band gap of a solid solution of GaN:ZnO is 2.58–2.76 eV, which showed visible-light-driven photocatalytic activity for complete water splitting. The absorbance spectrum of GaN:ZnO after coating with porphyrins showed a new band around 550–1100 nm, suggesting composites of porphyrin/GaN:ZnO formed successively. After optimizing the photocatalysts and GaN:ZnO, RhO_x_, NiO /porphyrin/IrO_2_-GaN:ZnO showed effective fluorescence quenching with a 0.94 ns half-life, which was significantly shorter than that with IrO_2_-GaN:ZnO (2.9 ns half-life). This result suggested that the energy of GaN:ZnO was readily transferred to the porphyrins. When the RhO_x_, NiO/porphyrin/IrO_2_-GaN:ZnO was irradiated with a combination of 300 nm and 600 nm wavelength light, efficient hydrogen (8.3 μmol/h) and oxygen (3.9 μmol/h) production were achieved, which was higher than that obtained without a porphyrin photocatalyst (hydrogen: 2.2 μmol/h, oxygen: 1.1 μmol/h), suggesting the Z-scheme-type photocatalytic reaction (Figure 11(d)) [153].

## 9. Summary

In this work, various organic–inorganic hybrid photocatalysts for hydrogen production by water splitting have been reviewed. The organic–inorganic hybrid photocatalyst system has many advantages: (i) the absorption range can be improved, even if the band gap of the semiconductor is > 3.0 eV, by using organic dye molecules that absorb light in the visible to near IR region; (ii) the HOMO–LUMO energy of the molecules can be adjusted by modifying their molecular structure; (iii) charge separation can be enhanced and surface modes can be modified; and (iv) it can be used for co-catalysts instead of noble metals. For dye-sensitized photocatalytic hydrogen production from water, the design of molecular structures is important for achieving visible-light-driven photocatalytic activity. Metal complexes such as ruthenium and donor–bridge–acceptor-type complexes have shown enhanced responses in the visible range. The anchoring group of the dye can affect its orientation on the photocatalyst, and this can increase the charge recombination lifetime. The longer charge recombination lifetime increases the number of electrons available for photosynthesis. Organic molecules can also be used to modify the surface of photocatalysts, which will prevent electron leakage due to charge recombination. Metal complexes have been effectively used as co-catalysts for hydrogen production. For example, platinum, cobalt, nickel and iron metal complexes have been used as reaction centres for hydrogen production, together with a combination of typical semiconductors such as TiO_2_, visible-light-responsive CdX (X = S, Se, or Te) and the organic photocatalyst C_3_N_4_. The combination of Z-scheme-type organic–inorganic photocatalysts has also been discussed. The Z-scheme-type system requires a p–n-type photocatalyst with a redox mediator. Both semiconductors and organic dyes can be selected in the visible light response system. The system could efficiently split water to produce hydrogen and oxygen, indicating effective charge separation in the system. Inorganic semiconductors coated by organic molecules can also be used for Z-scheme-type photocatalytic water splitting.

This review has listed organic materials that have high potential for use as visible-light-driven photocatalysts and co-catalysts. The organic materials can also be used in dye-sensitized-type systems for hydrogen production and Z-scheme-type systems for complete water splitting, indicating that the organic–inorganic system has potential for various applications, similar to inorganic composite systems.

Thus, the advantages of hydrogen production using a hybrid system have been demonstrated. However, designing organic molecules or systems to increase a hydrogen production rate beyond that obtained by natural systems is still a challenging issue. In addition, many hybrid organic sensitizer systems required sacrificial electron donor reagents as it is difficult to oxidize water by organic dye itself. Difficult choices about the specific type of organic hybrid system to be used as a photocatalyst also still remain. This flexible system can be used in other applications such as organic transformation systems. In the future, organic–inorganic hybrid materials will be used to build photocatalyst systems exhibiting higher efficiencies.

## Funding

This work was supported by a Grant-in-Aid for Science Research [grant numbers JP17H04888; JP17K19123] from the Ministry of Education, Culture, Sports, Science and Technology (MEXT). The author acknowledges support from I^2^CNER, funded by the World Premier International Research Centre Initiative (WPI), Ministry of Education, Culture, Sports, Science and Technology of Japan (MEXT), Japan.

## Disclosure statement

No potential conflict of interest was reported by the author.
